# Association between lower geriatric nutritional risk index and low cognitive functions in United States older adults: a cross-sectional study

**DOI:** 10.3389/fnut.2024.1483790

**Published:** 2024-11-13

**Authors:** Jiuling Liu, Melysze Deanne Oorloff, Adithya Nadella, Ning Zhou, Min Ye, Yifeng Tang, Yuanwei Wang

**Affiliations:** ^1^Department of Neurology, Nanjing BenQ Medical Center, The Affiliated BenQ Hospital of Nanjing Medical University, Nanjing, China; ^2^Nanjing Medical University, Nanjing, China; ^3^Department of Radiology, Affiliated Shuyang Hospital of Xuzhou Medical University, Shuyang, China; ^4^Department of Neurology, Affiliated Shuyang Hospital of Xuzhou Medical University, Shuyang, China

**Keywords:** geriatric nutritional risk index, cognitive functions, NHANES, older adults, United States

## Abstract

**Background:**

We aimed to explore the association between the Geriatric Nutritional Risk Index (GNRI) and the risk of low cognitive functions among older adults in the United States (US).

**Methods:**

Utilizing data from the National Health and Nutrition Examination Study (NHANES) database, a cross-sectional analysis was conducted. The GNRI served as a tool for evaluating the nutritional status of participants, who were categorized into two groups based on their initial GNRI scores: those with scores >98 indicating normal nutrition, and those with scores ≤98 indicating malnutrition. Cognitive function was assessed using the Consortium to Establish a Registry for Alzheimer’s disease word list learning test (CERAD W-L), the Digit Symbol Substitution Test (DSST), the Animal Fluency Test (AFT), and the composite-z score which was calculated by summing the z scores of individual tests, respectively. Weighted multiple logistic regression models were used to evaluate the association between GNRI and cognitive function. Interaction and stratified analyses were conducted.

**Results:**

Among a sample of 2,925 individuals aged 60 years or older, 51.3% were women. Among these individuals, 233 were identified as malnourished. Weighted multivariate logistic regression analyses indicated that individuals with malnutrition had an increased risk of low cognitive function, as evidenced by lower CERAD W-L scores (OR:1.68, 95%CI 1.19–2.36, *p* = 0.003), AFT scores (OR: 1.74, 95%CI 1.26–2.41, *p* = 0.009), DSST scores (OR:1.63, 95%CI 1.11–2.38, *p* = 0.012), or composite z-scores (OR:1.87, 95%CI 1.29–2.71, *p* = 0.001). According to the variables evaluated, the interaction effects between low GNRI level and the elderly and stroke in specific cognitive domains were significant (P _interaction_ < 0.05).

**Conclusion:**

Lower GNRI level is associated with significantly low cognitive function among older adults, particularly among those who have experienced a stroke or the elderly (aged 70 years and older) population.

## 1 Introduction

Along with the increasing global elderly population, the population suffering from age-related cognitive impairment is growing at a rapid rate (1). Cognitive impairment encompasses a spectrum of conditions that includes Alzheimer’s disease (AD), vascular dementia, and other types. According to statistics, up to 12 million people in the United States (US) suffer from cognitive impairment. By 2060, this is expected to be double the size it was in 2020 (2). This trend underscores the urgent public health significance of cognitive impairment on a global scale (3). On this note, nutrition has been recognized as a modifiable factor that plays a major role in cognitive function. It is imperative to understand the relationship between nutrition and cognition so strategies that prevent and manage cognitive decline can be improved.

Research has shown a high prevalence of malnutrition among older adults with cognitive impairment (4–6). The majority of studies have investigated this relationship through the utilization of the Mini Nutritional Assessment (MNA) (7, 8) or Mini Nutritional Assessment Short-Form (MNA-SF) (9, 10). It is important to acknowledge that both MNA and MNA-SF rely on subjective questionnaires to evaluate nutritional status, lacking objective biological markers that exhibit significant variability. A widely accepted definition of malnutrition is “a state of nutrition, which characterized by a deficiency or excess of energy, protein, and micronutrients resulting in measurable negative effects on tissue or body form (body shape, size, and composition), function, and clinical outcomes (11). The Geriatric Nutritional Risk Index (GNRI), developed by Bouillanne et al. (12), utilizes serum albumin levels, weight, and height as objective measures to evaluate malnutrition. It has been acknowledged as a straightforward and effective method for assessing the nutritional status of elderly individuals aged 65 years and older (13), and has demonstrated efficacy in various countries (14–16). Previous studies have shown that the GNRI is a better indicator of nutrition-related risk than albumin or BMI alone (17, 18). The GNRI has been identified as a more appropriate tool for assessing the nutritional status of elderly individuals compared to the MNA or MNA-SF (14, 19). While existing research has indicated a significant correlation between GNRI levels and cognitive impairment, these studies are few and limited to China (13, 20–22). There remains a lack of research investigating the relationship between GNRI and cognitive functions in older adults within the United States.

Therefore, the objective of our study was to examine the association between malnutrition, as determined by the GNRI, and low cognitive function in US adults aged 60 years and older using data from the National Health and Nutrition Examination Survey (NHANES) for the years 2011–2012 and 2013–2014.

## 2 Methods

### 2.1 Study design and participants

Participants from the NHANES were included in this cross-sectional study. NHANES is a biennial survey administered by the National Center for Health Statistics (NCHS) that gathers health examination data from a representative sample of the US population (23–25). This data is utilized for the analysis of the nation’s health and nutritional status. All NHANES data was de-identified, and participants provided written informed consent. The NCHS Research Ethics Committee granted approval for all study procedures (25). The Ethics Committee of BenQ Hospital at Nanjing Medical University determined that this secondary analysis of de-identified, publicly available data did not involve human subjects.

**Figure 1:**
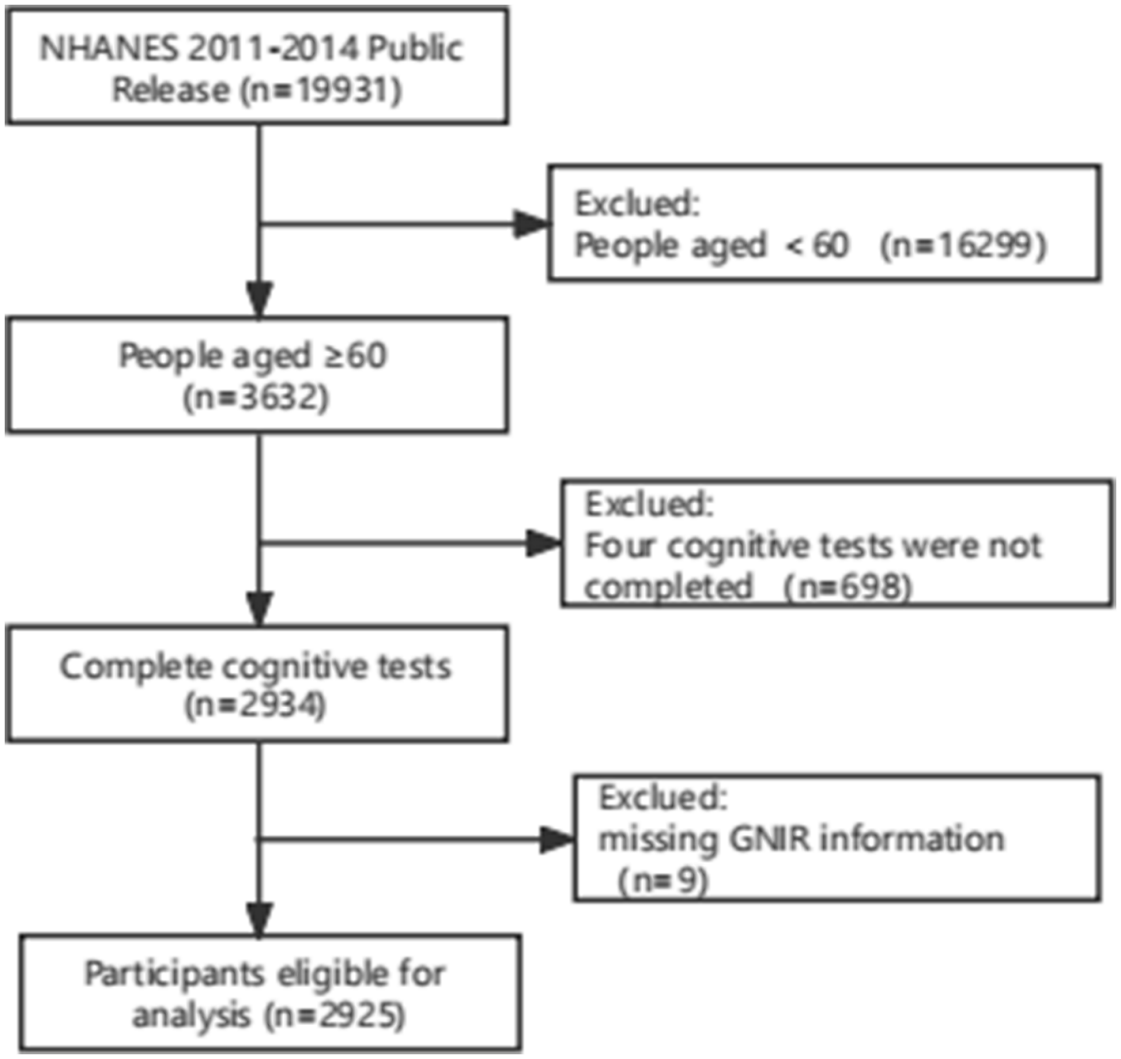
Flowcharts illustrating sample selection from NHANES 2011–2014. NHANES, the National Health and Nutrition Examination Survey.

The data of NHANES cycles were combined for analysis, resulting in a total of 19,931 participants. Only the 2011–2012 and 2013–2014 cycles contained both baseline information on the GNRI and cognitive testing among older adults. However, only individuals aged 60 years and older underwent cognitive testing, leading to the exclusion of 16,299 participants outside of this age range. After excluding individuals who did not complete all four cognitive tests, the sample size was further reduced to 2,934 participants. Subsequently, participants without GNRI information were excluded, resulting in a final sample size of 2,925 participants for analysis. The process of participant inclusion and exclusion is illustrated in Figure 1.

### 2.2 Group

In our study, the exposure of interest was the GNRI, utilized for the evaluation of nutritional status. The GNRI is calculated using an individual’s height (in meters), weight (in kilograms), ideal weight, and serum albumin levels (in g/L). The formula for calculating GNRI is as follows: GNRI = 1.489 × albumin (g/L) + 41.7 × (weight/ideal weight) (12), ideal weight = 22 × height (m) × height (m). In cases where the actual weight exceeded the ideal weight, the set weight/ideal weight was 1 (12, 26). Participants were divided into two groups according to their baseline GNRI levels: the normal nutrition group (GNRI ≥98) and the malnutrition group (GNRI <98). Based on previous studies, the GNRI has been shown to be valid and reliable (12, 22, 27).

### 2.3 Cognitive function tests

Cognitive tests were conducted at the start of private interviews that occurred face-to-face by trained interviewers. The NHANES uses the Consortium to Establish a Registry for Alzheimer’s Disease word list learning test (CERAD W-L), the Animal Fluency test (AFT), and the Digit Symbol Substitution test (DSST) to assess different cognitive domains, which were validated in previous publications (28, 29).

The CERAD W-L subtest assesses immediate and delayed learning ability for new verbal information, a component of the memory sub-domain (30). The test includes 3 consecutive learning trials (CERAD Trial 1 Recall, CERAD Trial 2 Recall, and CERAD Trial 3 Recall) and 1 delayed recall trial. During the learning trial phase, participants were required to read 10 unrelated words aloud and then were instructed to immediately recall as many words as possible. The order of the 10 words was different in each learning trial. The delayed word recall was performed after the completion of the other two cognitive tests (AFT and DSST). In this study, we summarized the score of the CERAD W-L Test immediate and delayed learning ability for new verbal information as the total CERAD W-L score. The score range of each trial was from 0 to 10, and the total CERAD W- L score is the sum of three consecutive learning trials and one delayed recall trial (31). The AFT is a measure of categorical verbal fluency, with scores ranging from 3 to 39, and assesses executive function (31, 32). Participants were asked to say the names of animals as many as possible during 1-min trials. The DSST, with scores ranging from 0 to 105, assesses psychometric speed and attention (29, 33, 34). Participants filled in an array of symbols that corresponded to specified digits during 120-s trials. Details on each cognitive test can be found in the 2011–2012 and 2013–2014 NHANES questionnaire data files. Based on methods previously published, we used means and standard deviations of the cognitive test scores to calculate test-specific z-scores for the CERAD W-L, the AFT, and the DSST, respectively (35, 36). Global cognition composite z-score was then calculated by averaging all test-specific z-scores. This composite z-score was then used to evaluate the overall cognitive function of the study participants. Higher scores on all measures indicate better cognitive performance.

Although there was no uniform definition of low cognitive function, previous studies have defined low cognitive function as falling within the lowest quartile on the cognitive tests (37–40). Therefore, based on methods previously published, we determined the cutoff at the 25th percentile, or lowest quartile, for each respective test (41). The cutoff values identified were as follows: for CERAD W-L scores was set at <20; for AFT scores was <13; for DSST scores was <33, and composite z-score was < −1.7. Based on these thresholds, participants were subsequently categorized into two groups: low cognitive function (LC) group and normal cognitive function (NC) group (37).

### 2.4 Covariates

We obtained variables by virtue of a standardized self-reported questionnaire including age, sex, race (Mexican American, non-Hispanic White, non-Hispanic Black and other race), marital status (married, widowed, divorced, separated, never married, and living with partner), poverty income ratio (PIR) and educational level (less than high school, high school, college or higher), smoking status, alcohol consumption, history of diseases [diabetes, hypertension, stroke, Parkinson’s disease (PD), cardiovascular disease (CVD)], moderate to vigorous work activity (yes/no), moderate to vigorous physical activity (yes/no), sleep duration, albumin level (≥35 g/L or < 35 g/L) and depression score from the NHANES database. Additionally, the family’s PIR was divided into two categories (<1 or ≥ 1) to reflect house income. Higher ratios indicate better financial conditions for families. In our study, participants were classified as having diabetes based on HbA1c levels of 6.5% or more and/or current use of insulin or diabetic pills. Hypertension was defined as having a SBP ≥140 mmHg or a DBP ≥ 90 mmHg and/or currently using antihypertensive medication. This study assessed depression using the 9-item Patient Health Questionnaire (PHQ-9). The scores of each item range from 0 (not at all) to 3 (nearly every day), yielding a maximum score of 27. Subjects with PHQ-9 score ≥ 10 were identified as depression (42). In our study, PD cases were characterized as taking any of the following PD-specific medications: benztropine, levodopa, carbidopa, methyldopa, ropinirole, entacapone, or amantadine (22, 43). Self-reported CVD included congestive heart failure, coronary heart disease, angina, and heart attack. During the in-person interview, participants of the National Health Interview Survey were requested to report their engagement in moderate- and vigorous-intensity physical activities. Trained medical personnel employed a series of questions, derived from the Global Physical Activity Questionnaire, to gather information regarding physical activities related to work, transportation, and leisure time. The activity classifications encompassed five types: vigorous work activity, vigorous leisure activity, moderate work activity, moderate leisure activity, and transportation-related activity (44). Participants of the National Health Interview Survey provided self-reported data on sleep duration during in-person interviews by responding to the question: “On average, how many hours of sleep do you obtain in a 24-h period?” Sleep duration of fewer than 7 h per day was classified as insufficient (45–47).

### 2.5 Statistical analysis

To account for the complicated design of NHANES, primary sampling units (clusters), stratification, and sample weights were accounted for in the analysis based on the NHANES analysis course. Since we merged data from both cycles (2011–2012 and 2013–2014), we utilized the original sample weights, WTMEC2YR, when divided by two as the new sample weights.

Participants were categorized into two groups by baseline GNRI levels, established by a previous study (48, 49): the group with normal nutrition (GNRI ≥98) and the group with malnutrition (GNRI <98). Numbers (N) and percentages (%) were expressed according to categorical variables, and the comparisons between groups were made using *χ*^2^ tests. We described continuous variables as weighted means (SE) or median (P_25_, P_75_) and categorical variables as weighted frequencies (%). One-way ANOVA was performed if the data were normally distributed, and when not, the Kruskal-Wallis test was used.

The study examined the association between baseline GNRI levels and cognitive function, utilizing total CERAD W-L score, AFT, DSST, and composite z-score as dichotomous variables to categorize participants into LC and NC groups. Weighted univariate and multivariate logistic regression models were employed to analyze the relationship. Three models were constructed: the first model was unadjusted, the second model was adjusted for age, sex, race, education, marital status, and PIR, and the third model was adjusted for all baseline variables. In addition, we used curve-fitting to visualize the relationship between GNRI and low cognitive function.

We also performed a sensitivity analysis by restricting participants without stroke and Parkinson’s disease. Additionally, we performed interaction and stratified analyses based on age categories (60–69, 70–79, and ≥ 80 years), gender (male/female), educational attainment (less than high school/high school/college/higher), PIR (<1 or ≥ 1), smoking habits (never/former/current), alcohol consumption, histories of disease (diabetes, hypertension, stroke, CVD, Parkinson, depression), and moderate to vigorous work activity. All statistical analyses were carried out using the R software package^1^ and EmpowerStats. *p* values less than 0.05 (2-sided) were considered significantly different.

## 3 Results

### 3.1 Study population characteristics

The weighted distribution of participants is described in Table 1. A total of 2,925 patients (1,425 male and 1,500 female) aged 60 years and older were eligible for this study. Of those, 45.6% (weighted proportion) were male. Based on the presence of nutritional status, participants were divided into two groups: the normal nutritional group (GNRI >98, *n* = 2,692) and the malnutrition group (GNRI ≤98, *n* = 233) following a previous study. Baseline participant characteristics stratified by baseline GNRI levels (> 98 and ≤ 98) were set out in Table 1. Compared to the normal nutritional group, participants in the malnutrition group were more likely to be older, had a higher proportion of non-Hispanic White; had a lower prevalence of moderate to vigorous work activity and a higher risk to develop comorbidities, such as hypertension and stroke (all *p* < 0.05). Participants who had malnutrition also scored lower in cognitive function, including the CERAD W-L, DSST, AFT and composite z-score.

**Table 1:** Weighted characteristics and measured data from NHANES 2011–2014 participants aged 60 years and over according to GNRI (High >98 Versus Low ≤98).

Variables	All	Nutritional status		*p*- value
		Normal (GNRI > 98)	Malnutrition (GNRI ≤ 98)	
Unweighted N	2,925	2,692	233	
Age, median (IQR)	69.2 (6.5)	69.0(6.6)	70.9 (6.9)	0.058
Age group, *n* (%)				0.047
60–69	1,462 (51.6)	1,366 (52.2)	96(42.8)	
70–79	930 (31.9)	855(32.0)	75(30.7)	
≥80	533 (16.5)	471(15.8)	62(26.5)	
Sex, *n* (%)				0.471
Male	1,425 (45.6)	1,307 (48.6)	118 (41.9)	
Female	1,500 (54.4)	1,385 (51.4)	115 (58.1)	
Race, *n* (%)				< 0.001
Mexican-American	257 (3.4)	242 (9)	15 (3.1)	
Non-Hispanic White	1,397 (79.5)	1,308 (48.6)	89 (68.8)	
Non-Hispanic Black	695 (8.4)	611 (22.7)	84 (15.9)	
Other Race	576 (8.7)	531 (19.7)	45 (12.0)	
Marital status, *n* (%)				0.639
Married	1,612 (62.2)	1,499 (55.7)	113 (58.2)	
Widowed	572 (17.0)	518 (19.3)	54 (21.7)	
Divorced	166 (4.3)	155 (5.8)	11 (3.2)	
Separated	415 (12.7)	380 (14.1)	35 (12.6)	
Never married	78 (1.2)	68 (2.5)	10 (1.5)	
Living with partner	78 (2.6)	70 (2.6)	8 (2.8)	
PIR (%)				0.059
<1	457 (9.1)	407 (16.5)	50 (13.2)	
≥1	2,220 (90.9)	2061 (83.5)	159 (86.8)	
Education, *n* (%)				0.405
Less than high school	744 (15.9)	667 (24.8)	77 (20.5)	
High school	684 (22.2)	636 (23.6)	48 (23.4)	
College or higher	1,494 (61.9)	1,387 (51.6)	107 (56.1)	
Smoking status, *n* (%)				0.265
never	1,440 (49.6)	1,332 (49.5)	108 (47.4)	
former	1,112 (39.4)	1,015 (37.7)	97 (44.5)	
now	371 (11.0)	343 (12.8)	28 (8.0)	
Alcohol consumption, *n* (%)				0.365
never	450 (13.0)	411 (15.6)	39 (15.1)	
former	801 (23.2)	725 (27.4)	76 (28.8)	
moderate	281 (11.7)	263 (10)	18 (10.2)	
middle	1,124 (46.1)	1,052 (39.8)	72 (40.1)	
heavy	209 (5.9)	192 (7.3)	17 (5.8)	
History of diseases, n (%)				
Diabetes, n (%)	971 (27.0)	901 (33.5)	70 (29.1)	0.467
Hypertension, n (%)	2081 (67.1)	1905 (70.8)	176 (75.7)	0.048
Stroke, n (%)	212 (6.8)	186 (6.9)	26 (12.2)	0.023
CVD, n (%)	642 (22.0)	590 (21.9)	52(22.3)	0.890
Parkinson, n (%)	52 (1.9)	46 (1.7)	6 (3.3)	0.264
Moderate to vigorous work activity, *n* (%)				0.007
No	2042 (65.5)	1860 (69.1)	182 (75.8)	
Yes	882 (34.5)	831 (30.9)	51 (24.2)	
Moderate to vigorous physical activity, *n* (%)				0.615
No	1,385 (47.4)	1,271 (47.2)	114 (48.9)	
Yes	1,540 (52.6)	1,421 (52.8)	119 (51.1)	
PHQ score, median (IQR)	1.0 (0,4)	1.0 (0,4)	2.0 (0,4)	0.057
Sleep duration (h), mean (SE)	7.1 ± 2.8	7.1 ± 2.9	7.1 ± 1.6	0.911
Albumin level < 0.001				
≥35 g/L	2,879 (98.4)	2,657 (98.7)	222 (95.3)	
<35 g/L	46 (1.6)	35 (1.3)	11 (4.7)	
DSST score, mean (SE)	52.1 (16.8)	52.6 (16.6)	44.0 (17.6)	<0.001
AFT score, mean (SE)	18.1 (5.7)	18.2 (5.6)	17.1 (6.6)	0.043
CERAD total score, mean (SE)	26.0 (6.4)	26.1 (6.4)	24.6 (6.9)	0.030
Composite z-score, median (IQR)	0.4 (−0.3, 1.1)	0.5 (−0.2, 1.1)	0.03 (−0.8, 0.6)	< 0.001

In the NHANES study, the reporting of age in single years for adults 80 years and older was determined to be a disclosure risk. All responses of participants aged 80 years and older are coded as ‘80’. Mean (SE), median (IQR), or *n* (%). SE, standard error of mean; IQR, interquartile range; PIR, poverty-income ratio; CVD, cardiovascular disease; DSST, Digit Symbol Substitution Test; AFT, Animal Fluency Test; CERAD, consortium to Establish a Registry for Alzheimer’s Disease; The composite-z score was calculated by summing the z scores [(test score - mean score)/SD] of the three individual tests.

Taking into account clinical significance and defining low cognitive function as the lowest quartile of the cognitive test according to the NHANES database (37), each cognitive function test score was divided into a binary variable (normal and low cognitive function) for further analysis. Regardless of the cognitive assessment method used, the proportion of all participants in the LC group with malnutrition was significantly higher than that in the NC group (*p* < 0.05) (Table 2).

**Table 2:** Presence of low cognitive function in adults ≥60 years old from NHANES 2011–2014 according to GNRI (High >98 Versus Low ≤98).

Outcome	All	Nutritional status		*p*- value
		Normal (GNRI > 98)	Malnutrition (GNRI ≤ 98)	
Unweighted *N*	2,925	2,692	233	
Total CERAD W-L score				
NC, *n* (%)	2,192 (80.5)	2050 (81.1)	142 (72.2)	0.016
LC, *n* (%)	733 (19.5)	642 (18.9)	91 (27.8)	
AFT score				
NC, *n* (%)	2039 (78.5)	1914 (79.3)	125 (67.7)	0.004
LC, *n* (%)	886 (21.5)	778 (20.7)	108 (32.3)	
DSST score				
NC, *n* (%)	2,166 (85.1)	2030 (86.0)	136 (71.3)	<0.001
LC, *n* (%)	759 (14.9)	662 (14.0)	97(28.7)	
Composite z-score				
NC, *n* (%)	2,180 (84.9)	2047 (85.9)	133 (69.4)	<0.001
LC, *n* (%)	745 (15.1)	645 (14.1)	100 (30.6)	

NC, normal cognitive function group; LC, low cognitive function group; DSST, Digit Symbol Substitution Test; AFT, Animal Fluency Test; CERAD, consortium to Establish a Registry for Alzheimer’s Disease; The composite-z score was calculated by summing the z scores ((test score - mean score)/SD) of the three individual tests. Low total CERAD W-L score, low AFT score, low DSST score and low comprehensive z score were, respectively, evaluated for low cognitive function.

### 3.2 Multiple logistic regression analyses of GNRI and cognitive functions

**Figure 2:**
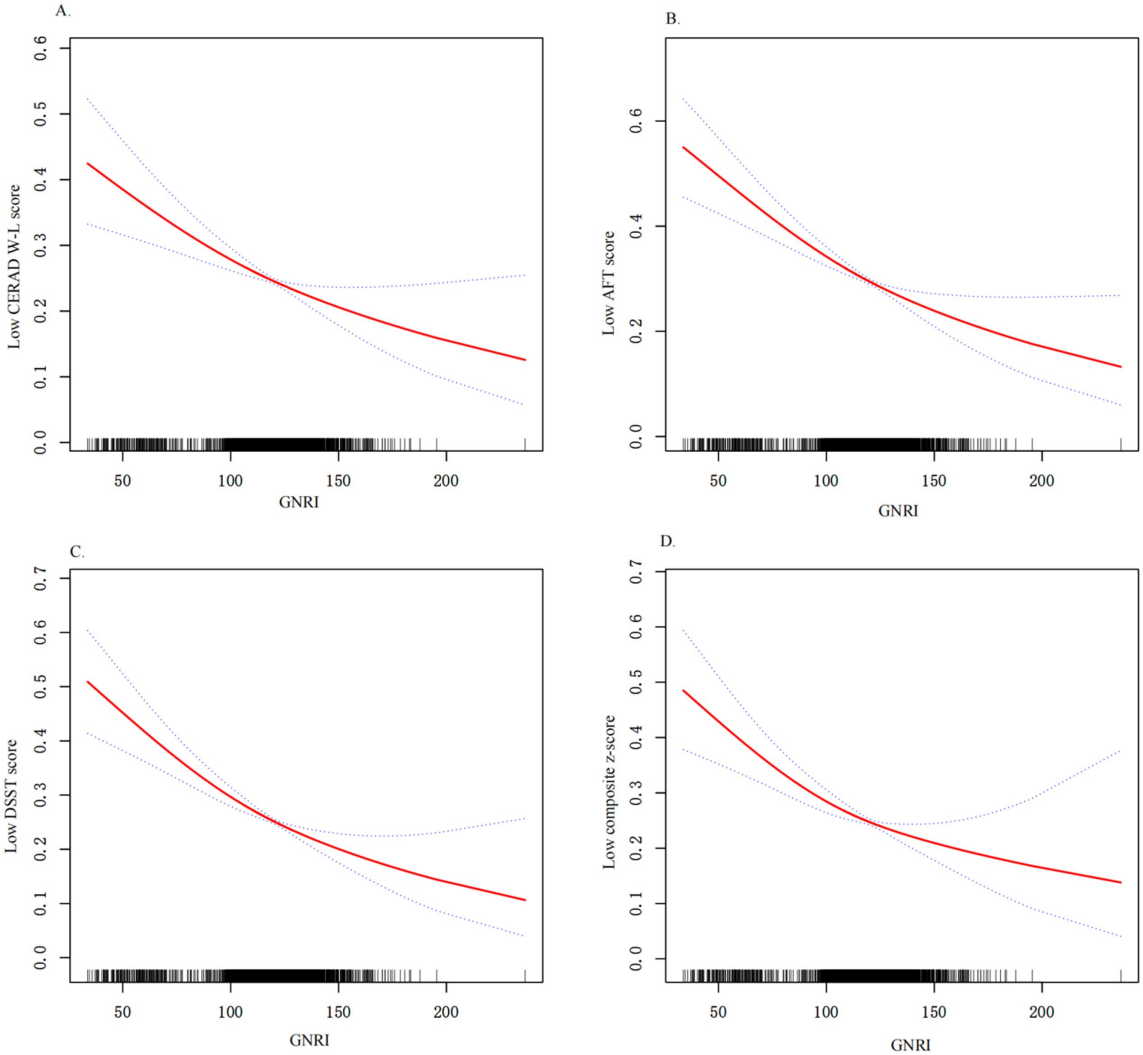
Relationship between GNRI and low cognitive functions. (A) Relationship between GNRI and low CERAD W-L score. (B) Relationship between GNRI and low AFT score; (C) Relationship between GNRI and low DSST score; (D) Relationship between GNRI and low composite z-score; All results were detected after adjusting for variables listed in Model 3. X-axis, the level of GNRI; Y-axis, the probability of low cognitive functions score; Solid line represents the smooth curve fit between variables. Bands present the 95% CI.

Next, we used weighted multiple logistic regression to assess the relationship between GNRI and cognitive functions. Three logistic regression models were generated to characterize the association between GNRI and cognitive functions (Table 3). The GNRI level ≥ 98 was taken as a reference in accordance with a previous study. In the crude model (unadjusted for covariates), the risk of low cognitive function was increased in subjects with malnutrition, as determined by total CERAD W- L score (OR: 2.05, 95%CI 1.55–2.70, and *p* < 0.001), AFT (OR: 2.13, 95%CI 1.62–2.79, and *p* < 0.001), DSST (OR: 2.19, 95%CI 1.66–2.88, and *p* < 0.001), or composite z-score (OR: 2.39, 95%CI 1.81–3.14, and *p* < 0.001), respectively, compared with the normal nutrition group. In model 1 (adjusted for age, sex, race, education, marital status, PIR), the results did not differ from those obtained in the crude analysis, and it indicated that malnutrition was associated with low cognitive functions. The results for placement in the LC group according to each test were as follows: total CERAD W- L score (OR: 1.77, 95%CI 1.28–2.44, and *p* = 0.001), AFT (OR: 1.78, 95%CI 1.30–2.42, and *p* < 0.001), DSST (OR: 1.65, 95%CI 1.15–2.36, and *p* = 0.006), or composite z-score (OR: 1.90, 95%CI: 1.34–2.70, and *p* < 0.001). In the fully adjusted model (adjusted all variables listed in Table 1), total CERAD W- L score (OR: 1.68, 95%CI 1.19–2.36, and *p* = 0.003), AFT (OR: 1.74, 95%CI 1.26–2.41, and *p* = 0.009), DSST (OR: 1.63, 95%CI 1.11–2.38, and *p* = 0.012), or composite z-score (OR: 1.87, 95%CI 1.29–2.71, and p = 0.001). When included as a continuous variable, yielding consistent results, as GNRI levels increase, the risk of low cognitive function is significantly decreased. These results indicated that GNRI was an independent risk factor for low cognitive functions. Additionally, smoothed curve fitting analyses indicated linear relationships between GNRI values and as determined by low total CERAD W- L score, low AFT score, low DSST score or composite z-score, respectively (Figure 2). Similar results were observed in sensitivity analyses (online Supplementary Table S1). When excluding individuals who suffered stroke (*n* = 205) or Parkinson’s disease (*n* = 52), GNRI (≤ 98) was still positively associated with low total CERAD W- L score (OR: 1.79; 95% CI: 1.24,2.59; *p* < 0.001), low AFT score (OR: 1.49; 95% CI: 1.05,2.11; *p* = 0.026), low DSST score (OR: 1.48; 95% CI: 1.01,2.16; *p* = 0.044) and low composite z-scores (OR: 1.53; 95% CI: 1.01,2.28; *p* = 0.046) in adjusted model 2. Moreover, the GNRI was analyzed as quartiles, indicating that a low GNRI level was associated with low cognitive functions in all participants (p for trend <0.001), yielding consistent results (online Supplementary Table S2).

**Table 3:** Unadjusted and adjusted associations between GNRI and Low cognitive function assessed by low CERAD W-L score, AFT score, DSST score and composite z-score.

Outcome (LC)	GNRI	Crude Model		Model 1		Model 2	
		OR (95%CI)	*p*- value	OR (95%CI)	*p*- value	OR (95%CI)	*p*- value
Low CERAD W-L score	GNRI (continuous)	0.99 (0.98,0.99)	<0.001	0.99 (0.98,0.99)	<0.001	0.99 (0.98,0.99)	<0.001
	GNRI >98	1 (Ref)		1 (Ref)		1 (Ref)	
	GNRI ≤98	2.05 (1.55,2.70)	<0.001	1.77 (1.28,2.44)	0.001	1.68 (1.19,2.36)	0.003
Low AFT score	GNRI (continuous)	0.99 (0.98,0.99)	<0.001	0.99 (0.98,0.99)	<0.001	0.99 (0.98,0.99)	<0.001
	GNRI >98	1 (Ref)		1 (Ref)		1 (Ref)	
	GNRI ≤98	2.13 (1.62,2.79)	<0.001	1.78 (1.30,2.42)	<0.001	1.74 (1.26,2.41)	0.009
Low DSST score	GNRI (continuous)	0.99 (0.98,0.99)	<0.001	0.99 (0.98,0.99)	<0.001	0.99 (0.98,0.99)	<0.001
	GNRI >98	1 (Ref)		1 (Ref)		1 (Ref)	
	GNRI ≤98	2.19 (1.66,2.88)	<0.001	1.65 (1.15,2.36)	0.006	1.63 (1.11,2.38)	0.012
Low Composite z-score	GNRI (continuous)	1.01 (1.01,1.02)	<0.001	1.01 (1.00,1.02)	<0.001	1.01(1.01,1.02)	<0.001
	GNRI >98	1 (Ref)		1 (Ref)		1 (Ref)	
	GNRI ≤98	2.39 (1.81,3.14)	<0.001	1.90(1.34,2.70)	<0.001	1.87 (1.29,2.71)	0.001

OR: Odd ratio; 95%CI: 95% confidence intervals; LC, low cognitive function group; In sensitivity analysis, GNRI were converted from continuous variables (1-unit increase) to categorical variables (> 98 or ≤ 98); Low CERAD W-L score, low AFT score, low DSST score and low Composite z-score were, respectively, evaluated for low cognitive function. Crude Model was adjusted for none. Model 1 was adjusted for age, sex, race, education, marital status, PIR. Model 2 was adjusted for age, sex, race, education, marital status, PIR, smoking status, drinking, work activity, depression, history of diseases (hypertension, stroke, diabetes, Parkinson, CVD).

Overall, our findings consistently demonstrate the positive relationship between GNRI levels and cognitive function scores across multiple measures, suggesting that higher GNRI levels are associated with better cognitive performance.

### 3.3 Subgroup analysis

**Figure 3:**
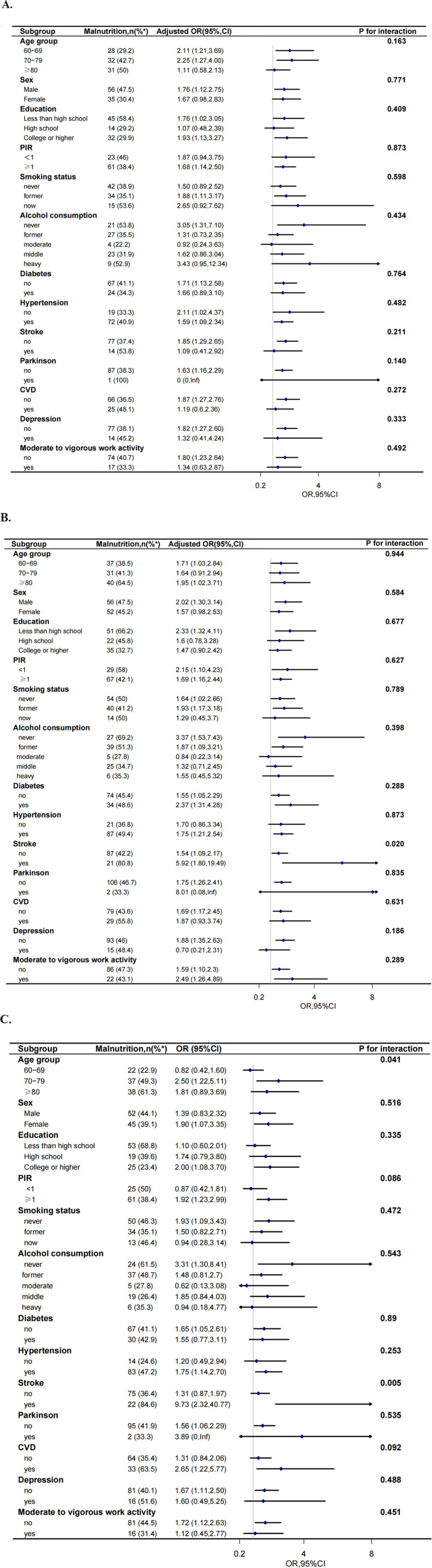
Effect size of GNRI on low CERAD W-L score (A), low AFT score (B), low DSST score (C) and composite z-score (D) in exploratory subgroups. The model was adjusted, if not stratified, for age, sex, race, education, marital status, PIR, smoking status, drinking, work activity, depression, history of diseases (hypertension, stroke, diabetes, Parkinson, CVD). (PIR, poverty-income ratio; CVD: cardiovascular disease); %^*^: the number of malnutrition as a percentage of the total number of participants.

To further decide how GNRI affects low cognitive functions, we performed a series of subgroup analyses, including age group (60–69, 70–79, and ≥ 80 years), gender, education level (less than high school, high school, college, or higher), PIR (<1or ≥1), smoking status, alcohol consumption, histories of disease (diabetes, hypertension, stroke, CVD, Parkinson, depression), and moderate to vigorous work activity. In participants given total CERAD W- L score, there was no interaction between GNRI and low cognitive functioning based on the above subgroups (all *p* for interaction >0.05). In participants given AFT scores, significant interactions were observed in the medical history of stroke status (*p* for interaction = 0.020). Lower GNRI was significant associated with low cognitive function (OR: 5.92, 95%CI 1.80–19.49) in participants with stroke. In participants given DSST scores, significant interactions were observed for age status (p for interaction = 0.041) and stroke subgroup (p for interaction = 0.005). Lower GNRI was significantly associated with low cognitive function in participants 70–79 years old (OR: 2.50, 95%CI 1.22–5.11) and older than 80 years old (OR: 1.81, 95% CI 0.89–3.69), respectively. Lower GNRI was significant associated with low cognitive function (OR: 9.73, 95%CI 2.32–40.77) in participants with stroke. In participants administered composite z-score, significant interactions were also observed for age status (*p* for interaction = 0.004). Lower GNRI was also associated with low cognitive function in participants 70–79 years old (OR: 2.84, 95%CI 1.41–5.71) and older than 80 years old (OR: 2.91, 95%CI 1.42–5.98) but not in participants with age less than 70 years old. And in participants with stroke, lower GNRI was significant associated with low cognitive function (OR: 11.34, 95%CI 2.58–49.86). In participants administered AFT score, DSST score, or composite z-score, no interactions were observed for sex, education level, smoking status, medical history of diabetes, Parkinson, CVD, depression, or hypertension (all *p* for interaction >0.05) (Figure 3).

## 4 Discussion

This study conducted a cross-sectional analysis to investigate the association between malnutrition, as assessed by the GNRI, and cognitive function. The results indicated that individuals identified as malnourished by the GNRI were more likely to experience low cognitive function events compared to those with normal nutrition. Participants with malnutrition had poorer cognitive performance, especially in the elderly (≥70 years old) and a medical history of stroke.

The GNRI was an objective, reliable, and simple tool for efficiently screening the nutritional status of elderly individuals, requiring only weight, height, and serum albumin levels to calculate the score and does not depend on the patient’s cooperation (12). Moreover, GNRI recognizes malnutrition with higher sensitivity compared to MNA or MNA-SF (14, 19). The correlation between GNRI and cognitive function was reported in previous studies (13, 20–22). These findings are partially consistent with our findings, in which lower GNRI levels were significantly associated with a higher risk of cognition impairment. In a study screening for malnutrition and frailty in 740 older adults, conducted at West China University in Sichuan, China, a high risk of cognitive impairment was found in individuals with lower GNRI scores (13). He et al. also discovered that the lower GNRI showed a significant correlation with AD in cognitive centers of neurology at Beijing Tiantan Hospital (20). A longitudinal cohort study in the Chinese elderly without cerebrovascular and mental illness diseases reported that the GNRI was significantly associated with cognitive function (21). Lee et al. (22), showed that a lower baseline GNRI level was associated with an increased likelihood of future Post-Stroke Cognitive Impairment development in ischemic stroke patients, which was in line with our study.

Examining subgroup analyses is essential for a scientific investigation. Unfortunately, the above published papers only performed subgroup analyses and they did not test for interactions, which inhibits the exploration of the true relationship between GNRI and low cognitive function. In our current study, we utilized age group (60–69, 70–79, and ≥ 80 years), gender, education level (less than high school, high school, college, or higher), PIR (<1or ≥1), smoking status, alcohol consumption, histories of disease (diabetes, hypertension, stroke, cardiovascular disease, Parkinson, depression), and moderate to vigorous work activity as stratification variables and conducted interaction tests. Sun et al. (21), found that a strong link was discovered between moderate-to-severe malnutrition and cognitive performance in individuals aged 90 years and older, as well as in illiterate women. Our findings suggest a strong link between malnutrition and low cognitive function in individuals aged 70 years and older. In the above study, the average age of the study population was 85 years old, with the average age of their moderately and severely malnourished cohort being over 90 years. In contrast, the average age of our study population was about 69 years. This phenomenon could be attributed to differences in the study population and research design, which might have contributed to this result. This suggests a similar trend of increasing risk of malnutrition and decline in cognitive performance with increasing age. Hence, we further explored this age-related phenomenon and suggested that the lack of a significant correlation between GNRI and cognitive decline in participants under 70 years of age may be attributable to several factors. For example, younger individuals may have a greater cognitive reserve, which can buffer against cognitive decline (50, 51). Research suggests that factors such as education, mental stimulation, and social engagement play significant roles in maintaining cognitive function in younger populations (52–54). Additionally, participants under 70 years old may exhibit a wider variability in health status. Many individuals in this age group might not yet exhibit the phenotypic expressions of cognitive decline, leading to a dilution of the GNRI-cognitive decline relationship. The GNRI may have a more pronounced effect in older adults due to age-related changes in metabolism, nutritional needs, and the impact of malnutrition on cognitive function (55, 56). This finding may be explained by the observed differences between older and younger individuals. However, the exact mechanisms of these age-related changes require further investigation.

In addition, this study also found that the history of stroke is one of the main factors that affects cognitive functions and malnutrition. Lee et al. (22), reported that a lower GNRI was associated with post-stroke cognitive impairment, which was consistent with this study. Several reasons may explain this phenomenon, such as malnutrition negatively impacting brain plasticity and hindering proper protein synthesis and glucose utilization in the ischemic penumbra (57). This can result in a more severe stroke and worsening symptoms. Malnutrition was found to be linked to a higher risk of white matter high signal, microbleeds, and medial temporal lobe atrophy in individuals with mild cognitive impairment or dementia (58). Additionally, stroke survivors may experience dysphagia or respiratory infections, which can further worsen malnutrition. The association between malnutrition and cognitive function is unclear, but the GNRI validated the use of serum albumin and weight reduction in this examination to explore the connection instead of relying solely on a solitary biomarker. However, the exact role of the age, and history of stroke in mediating the relationship between malnutrition and low cognitive function likelihood still needs to be confirmed by large-scale studies.

This study has several strengths. Firstly, this study used a nationally representative sample of the US population based on the NHANES database. Secondly, we also used appropriate NHANES sample weights to analyze the data and used different cognitive tests, ensuring that the results were reliable and generalizable. In addition, we employed sensitivity analyses and subgroup analysis models to improve further the reliability of our evaluation of the relationship between the GNRI and low cognitive functions.

However, it is important to acknowledge the limitations of this study. First, the cross-sectional design cannot provide causality. Secondly, the use of baseline GNRI data from the NHANES study may underestimate the relationship between GNRI and cognitive impairment due to the lack of long-term follow-up data. Thirdly, the generalizability of these findings is limited as the analysis primarily includes individuals aged 60 and above from the United States. Fourthly, the database from NHANES 2012–2014 does not distinguish between types of cognitive impairment. And this may affect the clinical significance of GNRI. Future research should consider conducting larger cohort studies in diverse populations to further explore these associations.

## 5 Conclusion

This study suggested that a low GNRI level is an independent risk factor for low cognitive function among US adults aged 60 years or older. This was especially prevalent among the stroke population and the elderly in the DSST and psychometric speed and attention domains. Further cohort studies are required to clarify the cause-and-effect relationship between nutrition and cognitive impairment.

## Data Availability

The original contributions presented in the study are included in the article/Supplementary material, further inquiries can be directed to the corresponding author/s.

## Footnotes

^1^http://www.R-project.org, The R Foundation

## References

[1] Wang H Lv Y Ti G Ren G. Association of low-carbohydrate-diet score and cognitive performance in older adults: National Health and nutrition examination survey (NHANES). BMC Geriatr. (2022) 22:983. doi: 10.1186/s12877-022-03607-1, PMID: 36539697 PMC9764565

[2] Rajan KB Weuve J Barnes LL McAninch EA Wilson RS Evans DA. Population estimate of people with clinical Alzheimer’s disease and mild cognitive impairment in the United States (2020-2060). Alzheimers Dement. (2021) 17:1966–75. doi: 10.1002/alz.12362, PMID: 34043283 PMC9013315

[3] Jeremic D Jiménez-Díaz L Navarro-López JD. Targeting epigenetics: a novel promise for Alzheimer’s disease treatment. Ageing Res Rev. (2023) 90:102003. doi: 10.1016/j.arr.2023.102003 37422087

[4] Volkert D Chourdakis M Faxen-Irving G Frühwald T Landi F Suominen MH. ESPEN guidelines on nutrition in dementia. Clin Nutr Edinb Scotl. (2015) 34:1052–73. doi: 10.1016/j.clnu.2015.09.004, PMID: 26522922

[5] Doorduijn AS de van der Schueren M van de Rest O de Leeuw FA Hendriksen HMA Teunissen CE. Nutritional status is associated with clinical progression in Alzheimer’s disease: the NUDAD project. J Am Med Dir Assoc. (2023) 24:638–644.e1. doi: 10.1016/j.jamda.2020.10.020, PMID: 33239240

[6] Xu Lou I Ali K Chen Q. Effect of nutrition in Alzheimer’s disease: a systematic review. Front Neurosci. (2023) 17:1147177. doi: 10.3389/fnins.2023.1147177, PMID: 37214392 PMC10194838

[7] Assis APM De Oliveira BTN Gomes AL Soares ADN Guimarães NS Gomes JMG. The association between nutritional status, advanced activities of daily living, and cognitive function among Brazilian older adults living in care homes. Geriatr Nurs. (2020) 41:899–904. doi: 10.1016/j.gerinurse.2020.06.014, PMID: 32653259

[8] Mantzorou M Vadikolias K Pavlidou E Serdari A Vasios G Tryfonos C. Nutritional status is associated with the degree of cognitive impairment and depressive symptoms in a Greek elderly population. Nutr Neurosci. (2020) 23:201–9. doi: 10.1080/1028415X.2018.1486940, PMID: 29914306

[9] HsuY-HChouM-YChuC-SLiaoM-CWangY-CLinY-T. Predictive effect of malnutrition on long-term clinical outcomes among older men: a prospectively observational cohort study. J Nutr Health Aging. (2019) 23:876–82. doi: 10.1007/s12603-019-1246-231641739

[10] Yu W Yu W Liu X Wan T Chen C Xiong L. Associations between malnutrition and cognitive impairment in an elderly Chinese population: an analysis based on a 7-year database. Psychogeriatrics. (2021) 21:80–8. doi: 10.1111/psyg.12631 33207393

[11] Agarwal E Miller M Yaxley A Isenring E. Malnutrition in the elderly: a narrative review. Maturitas. (2013) 76:296–302. doi: 10.1016/j.maturitas.2013.07.013 23958435

[12] Bouillanne O Morineau G Dupont C Coulombel I Vincent JP Nicolis I. Geriatric nutritional risk index: a new index for evaluating at-risk elderly medical patients. Am J Clin Nutr. (2005) 82:777–83. doi: 10.1093/ajcn/82.4.777, PMID: 16210706

[13] Zhao Y Lin T Hou L Zhang M Peng X Xie D. Association between geriatric nutritional risk index and frailty in older hospitalized patients. Clin Interv Aging. (2021) 16:1241–9. doi: 10.2147/CIA.S313827, PMID: 34234424 PMC8254179

[14] DiMaria-Ghalili RA. Integrating nutrition in the comprehensive geriatric assessment. Nutr Clin Pract. (2014) 29:420–7. doi: 10.1177/0884533614537076 24993586

[15] Komatsu M Okazaki M Tsuchiya K Kawaguchi H Nitta K. Geriatric nutritional risk index is a simple predictor of mortality in chronic hemodialysis patients. Blood Purif. (2015) 39:281–7. doi: 10.1159/000381798, PMID: 25925239

[16] Cereda E Pedrolli C. The geriatric nutritional risk index. Curr Opin Clin Nutr Metab Care. (2009) 12:1–7. doi: 10.1097/MCO.0b013e3283186f59 19057180

[17] Eaton WW Bilker W Haro JM Herrman H Mortensen PB Freeman H. Long-term course of hospitalization for schizophrenia: part II. Change with passage of time. Schizophr Bull. (1992) 18:229–41. doi: 10.1093/schbul/18.2.229 1621070

[18] Abd-El-Gawad WM Abou-Hashem RM El Maraghy MO Amin GE. The validity of geriatric nutrition risk index: simple tool for prediction of nutritional-related complication of hospitalized elderly patients. Comparison with mini nutritional assessment. Clin Nutr. (2014) 33:1108–16. doi: 10.1016/j.clnu.2013.12.005, PMID: 24418116

[19] Abd Aziz NAS Mohd Fahmi Teng NI Kamarul ZM. Geriatric nutrition risk index is comparable to the mini nutritional assessment for assessing nutritional status in elderly hospitalized patients. Clin Nutr ESPEN. (2019) 29:77–85. doi: 10.1016/j.clnesp.2018.12.002, PMID: 30661705

[20] He M Lian T Liu Z Li J Qi J Li J. An investigation into the potential association between nutrition and Alzheimer’s disease. Front Nutr. (2024) 11:1306226. doi: 10.3389/fnut.2024.1306226, PMID: 38515521 PMC10955128

[21] Sun B Zhao Y Lu W Chen Y. The relationship of malnutrition with cognitive function in the older Chinese population: evidence from the Chinese longitudinal healthy longevity survey study. Front Aging Neurosci. (2021) 13:159. doi: 10.3389/fnagi.2021.766159, PMID: 34880747 PMC8645828

[22] Lee M Lim J-S Kim Y Lee JH Kim C-H Lee S-H. Association between geriatric nutritional risk index and post-stroke cognitive outcomes. Nutrients. (2021) 13:1776. doi: 10.3390/nu13061776, PMID: 34070955 PMC8224551

[23] Elnakib S Vecino-Ortiz AI Gibson DG. A novel score for mHealth apps to predict and prevent mortality: further validation and adaptation to the US population using the US National Health and nutrition examination survey data set. J Med Internet Res. (2022) 24:e36787. doi: 10.2196/36787 35483022 PMC9240932

[24] Saint-Maurice PF Graubard BI Troiano RP Berrigan D Galuska DA Fulton JE. Estimated number of deaths prevented through increased physical activity among US adults. JAMA Intern Med. (2022) 182:349–52. doi: 10.1001/jamainternmed.2021.7755, PMID: 35072698 PMC8787676

[25] Zhou L. Association of vitamin B2 intake with cognitive performance in older adults: a cross-sectional study. J Transl Med. (2023) 21:870. doi: 10.1186/s12967-023-04749-5, PMID: 38037028 PMC10691015

[26] Tuan CH. Gender selection and fertility regulation in the process of family building in China. Chin J Popul Sci. (1992) 4:33–54. PMID: 12286124

[27] Li L Wang H Yang J Jiang L Yang J Wu H. Geriatric nutritional risk index predicts prognosis after hepatectomy in elderly patients with hepatitis B virus-related hepatocellular carcinoma. Sci Rep. (2018) 8:12561. doi: 10.1038/s41598-018-30906-8, PMID: 30135506 PMC6105611

[28] CFQ_G. Available at: https://wwwn.cdc.gov/Nchs/Nhanes/2011-2012/CFQ_G.htm (Accessed October 6, 2024).

[29] Rosano C Perera S Inzitari M Newman AB Longstreth WT Studenski S. Digit symbol substitution test and future clinical and subclinical disorders of cognition, mobility and mood in older adults. Age Ageing. (2016) 45:687–94. doi: 10.1093/ageing/afw116, PMID: 27496932 PMC5027641

[30] Fillenbaum GG van Belle G Morris JC Mohs RC Mirra SS Davis PC. Consortium to establish a registry for Alzheimer’s disease (CERAD): the first twenty years. Alzheimers Dement. (2008) 4:96–109. doi: 10.1016/j.jalz.2007.08.005, PMID: 18631955 PMC2808763

[31] Frith E Shivappa N Mann JR Hébert JR Wirth MD Loprinzi PD. Dietary inflammatory index and memory function: population-based national sample of elderly Americans. Br J Nutr. (2018) 119:552–8. doi: 10.1017/S0007114517003804, PMID: 29361990 PMC5839966

[32] Cardoso BR Hare DJ Macpherson H. Sex-dependent association between selenium status and cognitive performance in older adults. Eur J Nutr. (2021) 60:1153–9. doi: 10.1007/s00394-020-02384-0, PMID: 32918622

[33] Prokopidis K Giannos P Ispoglou T Witard OC Isanejad M. Dietary Fiber intake is associated with cognitive function in older adults: data from the National Health and nutrition examination survey. Am J Med. (2022) 135:e257–62. doi: 10.1016/j.amjmed.2022.03.022, PMID: 35367443

[34] Golub JS Brickman AM Ciarleglio AJ Schupf N Luchsinger JA. Association of Subclinical Hearing Loss with Cognitive Performance. JAMA Otolaryngol Head Neck Surg. (2020) 146:57–67. doi: 10.1001/jamaoto.2019.3375, PMID: 31725853 PMC6865840

[35] Li W Li S Shang Y Zhuang W Yan G Chen Z. Associations between dietary and blood inflammatory indices and their effects on cognitive function in elderly Americans. Front Neurosci. (2023) 17:1117056. doi: 10.3389/fnins.2023.1117056, PMID: 36895419 PMC9989299

[36] Yen F-S Wang S-I Lin S-Y Chao Y-H Wei JC-C. The impact of heavy alcohol consumption on cognitive impairment in young old and middle old persons. J Transl Med. (2022) 20:155. doi: 10.1186/s12967-022-03353-3, PMID: 35382817 PMC8981936

[37] Fan Y Liu W Chen S Li M Zhao L Wu C. Association between high serum tetrahydrofolate and low cognitive functions in the United States: a cross-sectional study. J Alzheimers Dis. (2022) 89:163–79. doi: 10.3233/JAD-220058, PMID: 35871329

[38] ArabLAngA. A crosectional study of the association between walnut consumption and cognitive function among adult US populations represented in NHANES. J Nutr Health Aging. (2015) 19:284–90. doi: 10.1007/s12603-014-0569-225732213

[39] Dj B Ea K Ca T Lc M. Cognitive performance in adults aged 60 and over: National Health and nutrition examination survey, 2011-2014. Natl Health Stat Rep. (2019) 126:1–23. 31751207

[40] Liu L Qiao S Zhuang L Xu S Chen L Lai Q. Choline intake correlates with cognitive performance among elder adults in the United States. Behav Neurol. (2021) 2021:2962245–11. doi: 10.1155/2021/2962245, PMID: 34745383 PMC8570899

[41] Geng R Zhang Y Liu M Deng S Ding J Zhong H. Elevated serum uric acid is associated with cognitive improvement in older American adults: a large, population-based-analysis of the NHANES database. Front Aging Neurosci. (2022) 14:1024415. doi: 10.3389/fnagi.2022.1024415, PMID: 36570535 PMC9772611

[42] Kroenke K Spitzer RL Williams JB. The PHQ-9: validity of a brief depression severity measure. J Gen Intern Med. (2001) 16:606–13. doi: 10.1046/j.1525-1497.2001.016009606.x, PMID: 11556941 PMC1495268

[43] Fan Y Zhang Y Li J Liu Y Chang H Jiang Y. Association between healthy eating index-2015 and various cognitive domains in US adults aged 60 years or older: the National Health and nutrition examination survey (NHANES) 2011-2014. BMC Public Health. (2021) 21:1862. doi: 10.1186/s12889-021-11914-2, PMID: 34654401 PMC8520277

[44] Loprinzi PD. Dose-response association of moderate-to-vigorous physical activity with cardiovascular biomarkers and all-cause mortality: considerations by individual sports, exercise and recreational physical activities. Prev Med. (2015) 81:73–7. doi: 10.1016/j.ypmed.2015.08.014, PMID: 26307435

[45] Zhang Q Zhang M Chen Y Cao Y Dong G. Nonlinear relationship of non-high-density lipoprotein cholesterol and cognitive function in American elders: a cross-sectional NHANES study (2011-2014). J Alzheimers Dis. (2022) 86:125–34. doi: 10.3233/JAD-215250, PMID: 35001890

[46] Sheehan CM Frochen SE Walsemann KM Ailshire JA. Are U.S. adults reporting less sleep?: findings from sleep duration trends in the National Health Interview Survey, 2004-2017. Sleep. (2019) 42:zsy221. doi: 10.1093/sleep/zsy221, PMID: 30452725 PMC6941709

[47] Yin S Wang J Bai Y Yang Z Cui J Wang J. Association between sleep duration and kidney stones in 34 190 American adults: a cross-sectional analysis of NHANES 2007-2018. Sleep. Health. (2022) 8:671–7. doi: 10.1016/j.sleh.2022.08.003 36216750

[48] Huang W Xiao Y Wang H Li K. Association of geriatric nutritional risk index with the risk of osteoporosis in the elderly population in the NHANES. Front Endocrinol. (2022) 13:965487. doi: 10.3389/fendo.2022.965487, PMID: 36523597 PMC9744963

[49] Tkachenko SS Rutskiĭ VV Tikhilov RM Vovchenko VI. Normalization of bone regeneration by oxygen barotherapy. Vestn Khir Im I I Grek. (1988) 140:97–100. PMID: 3407095

[50] Stern Y. Cognitive reserve in ageing and Alzheimer’s disease. Lancet Neurol. (2012) 11:1006–12. doi: 10.1016/S1474-4422(12)70191-6, PMID: 23079557 PMC3507991

[51] Shea TB Remington R. Cognitive improvement in healthy older adults can parallel that of younger adults following lifestyle modification: support for cognitive reserve during aging. J Alzheimers Dis Rep. (2018) 2:201–5. doi: 10.3233/ADR-180056, PMID: 30480262 PMC6218155

[52] Lee S Kawachi I Berkman LF Grodstein F. Education, other socioeconomic indicators, and cognitive function. Am J Epidemiol. (2003) 157:712–20. doi: 10.1093/aje/kwg042 12697575

[53] Du C Miyazaki Y Dong X Li M. Education, social engagement, and cognitive function: a cross-lagged panel analysis. J Gerontol B Psychol Sci Soc Sci. (2023) 78:1756–64. doi: 10.1093/geronb/gbad088, PMID: 37294899 PMC10561888

[54] Murakami K Kuriyama S Hashimoto H. Economic, cognitive, and social paths of education to health-related behaviors: evidence from a population-based study in Japan. Environ Health Prev Med. (2023) 28:9. doi: 10.1265/ehpm.22-00178 36709974 PMC9884565

[55] Meydani M. Nutrition interventions in aging and age-associated disease. Ann N Y Acad Sci. (2001) 928:226–35. doi: 10.1111/j.1749-6632.2001.tb05652.x 11795514

[56] Bourdel-Marchasson I Laksir H Puget E. Interpreting routine biochemistry in those aged over 65 years: a time for change. Maturitas. (2010) 66:39–45. doi: 10.1016/j.maturitas.2010.02.004 20197224

[57] Aquilani R Sessarego P Iadarola P Barbieri A Boschi F. Nutrition for brain recovery after ischemic stroke: an added value to rehabilitation. Nutr Clin Pract. (2011) 26:339–45. doi: 10.1177/0884533611405793 21586419

[58] de van der Schueren MAE Lonterman-Monasch S van der Flier WM Kramer MH Maier AB Muller M. Malnutrition and risk of structural brain changes seen on magnetic resonance imaging in older adults. J Am Geriatr Soc. (2016) 64:2457–63. doi: 10.1111/jgs.14385, PMID: 27792245

