# Supplementary figures and images for: Association between lower geriatric nutritional risk index and low cognitive functions in United States older adults: a cross-sectional study
Source: Front Nutr. 2024 Nov 13;11:1483790. doi: 10.3389/fnut.2024.1483790 (PMC11609906; doi:10.3389/fnut.2024.1483790)

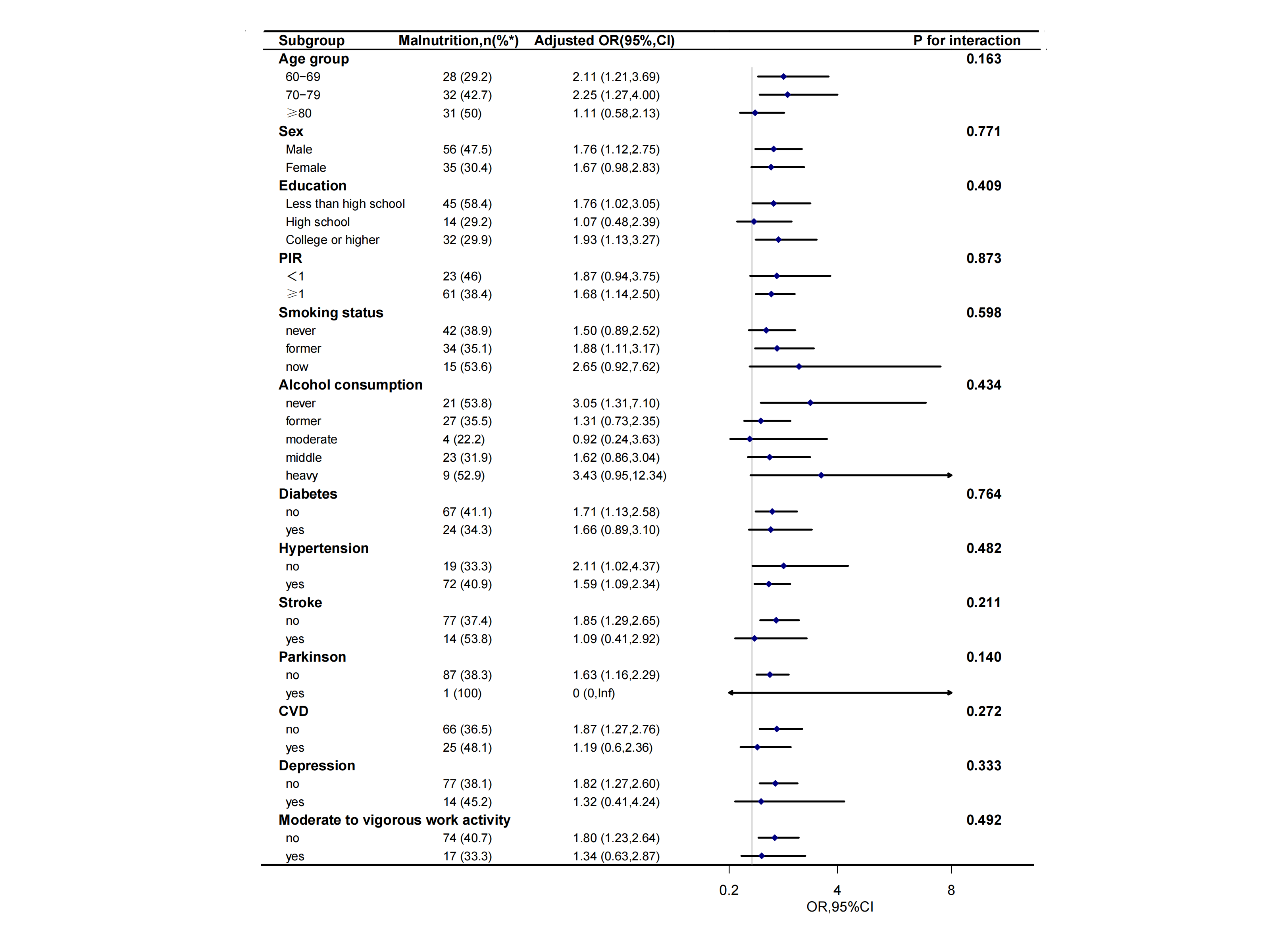

Supplement: Supplementary file 1 [file Image_1.TIF]

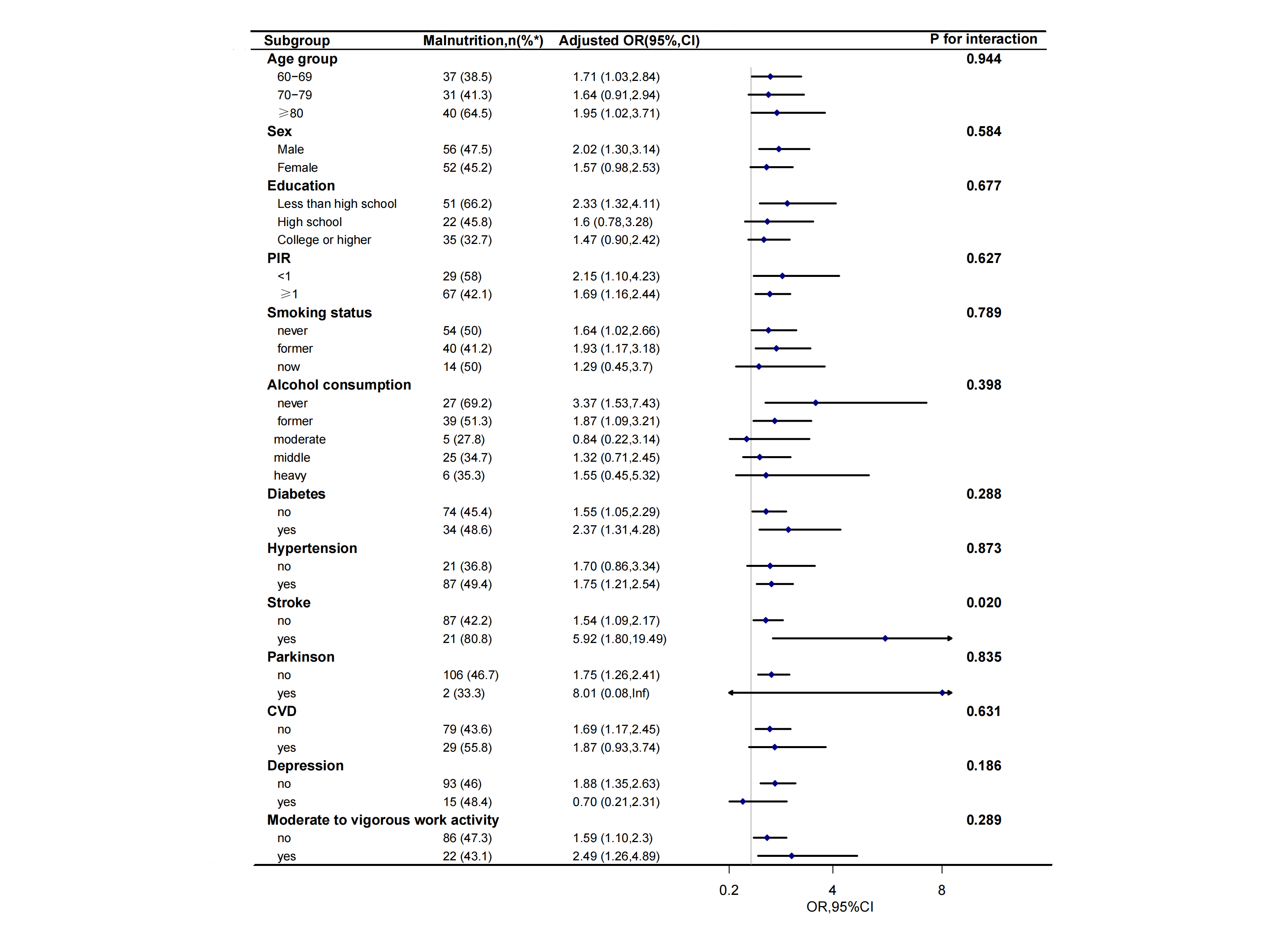

Supplement: Supplementary file 2 [file Image_2.TIF]

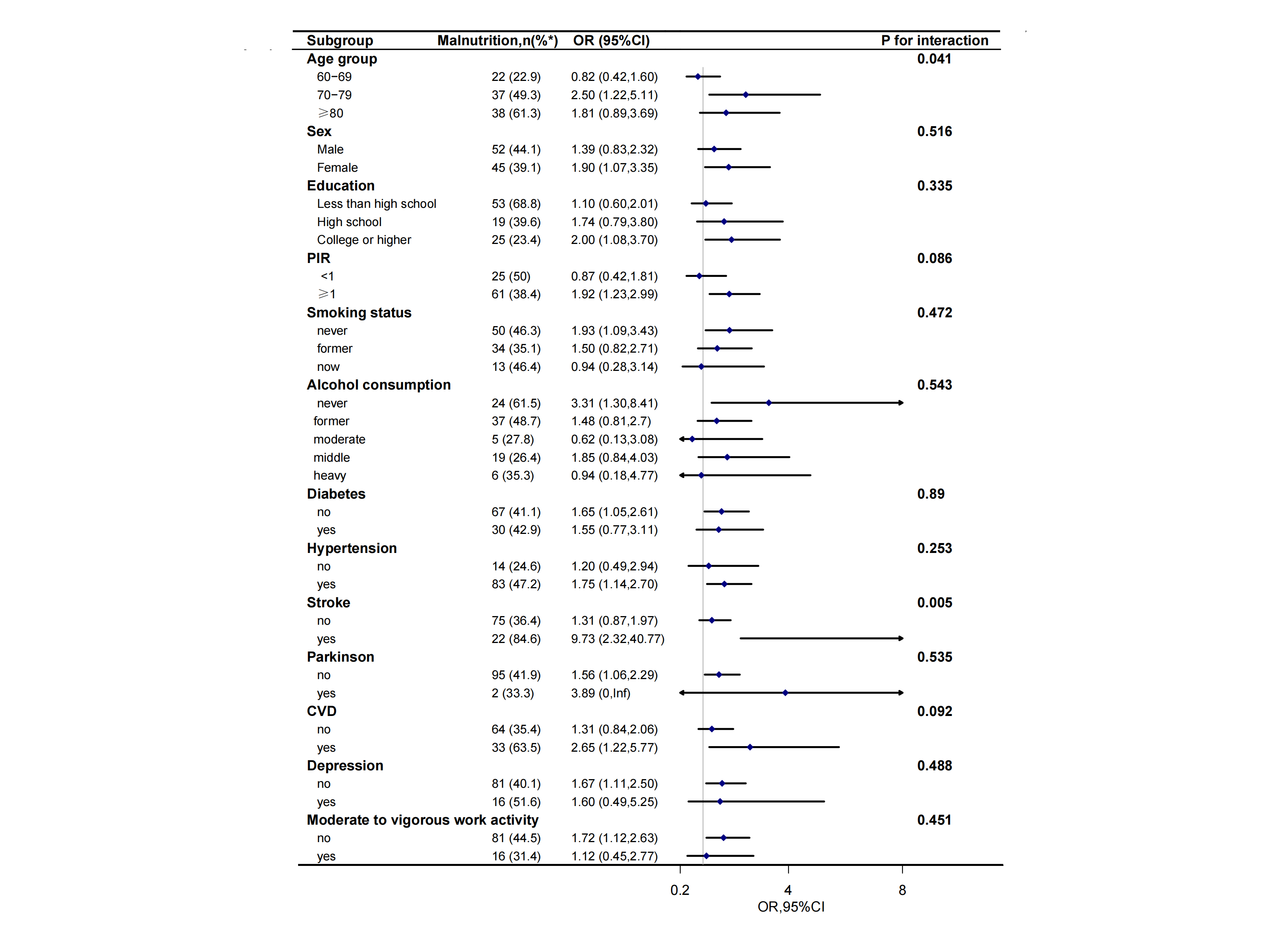

Supplement: Supplementary file 3 [file Image_3.TIF]

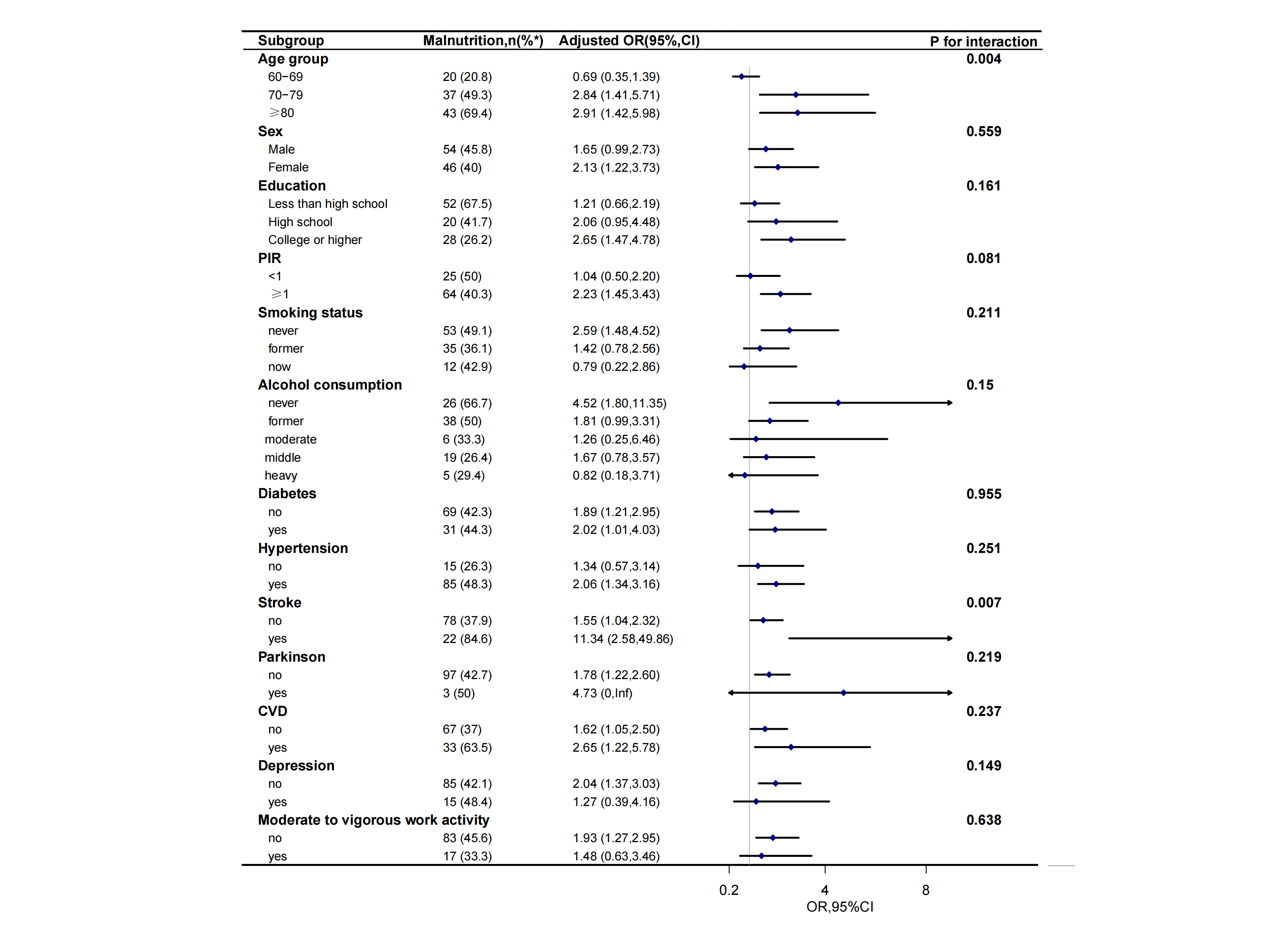

Supplement: Supplementary file 4 [file Image_4.TIF]
