# Supplementary material for: Association between lower geriatric nutritional risk index and low cognitive functions in United States older adults: a cross-sectional study
Source: Front Nutr. 2024 Nov 13;11:1483790. doi: 10.3389/fnut.2024.1483790 (PMC11609906; doi:10.3389/fnut.2024.1483790)
Supplement: Supplementary file 5 [file Table_1.doc]

**supplement Table 1. Unadjusted and Adjusted Associations Between GNRI and Low cognitive function assessed by low CERAD total score, AFT score, DSST score and Composite z-score, among patients without history of stroke or Parkinson's disease (n= 2668).**

| **Outcome (LC)** | **GNRI** | **Total/Event**  **N** | **Crude Model** | **Model 1** | **Model 2** |
| --- | --- | --- | --- | --- | --- |
|  |  |  | **OR (95%CI) *P* value** | **OR (95%CI) *P* value** | **OR (95%CI) *P* value** |
| **Low CERAD total score** | | | | | |
|  | GNRI > 98 | 2466/560 | 1(Ref) | 1(Ref) | 1(Ref) |
|  | GNRI ≤ 98 | 202/74 | 1.98(1.46,2.68) <0.001 | 1.81(1.27,2.58) <0.001 | 1.79(1.24,2.59) <0.001 |
| **Low AFT score** |  |  |  |  |  |
|  | GNRI > 98 | 2466/697 | 1(Ref) | 1(Ref) | 1(Ref) |
|  | GNRI ≤ 98 | 202/85 | 1.87(1.39,2.50) <0.001 | 1.57(1.15,2.14)0.004 | 1.49(1.05,2.11)0.026 |
| **Low DSST score** |  |  |  |  |  |
|  | GNRI > 98 | 2466/575 | 1(Ref) | 1(Ref) | 1(Ref) |
|  | GNRI ≤ 98 | 202/74 | 1.91(1.41,2.58) <0.001 | 1.55(1.09,2.21)0.014 | 1.47(0.99,2.15)0.053 |
| **Low Composite z-score** | | | | | |
|  | GNRI > 98 | 2466/562 | 1(Ref) | 1(Ref) | 1(Ref) |
|  | GNRI ≤ 98 | 202/76 | 2.05(1.52,2.77) <0.001 | 1.69(1.17,2.47)0.005 | 1.52(1.01,2.28)0.047 |

Note: OR, Odds Ratio; 95%CI, 95% confidence intervals; LC, low cognitive function.

Crude Model was adjusted for none.

Model 1 was adjusted for age, sex, race, education, marital status, PIR.

Model 2 was adjusted for age, sex, race, education, marital status, PIR, smoking status, drinking, work activity, depression, history of diseases (hypertension, diabetes, CVD).

**Supplement Table 2.** Weighted multiple logistic regressions analysis between GNRI and cognitive functions assessed by the composite z-score, AF, DSST, CERAD-DR and CERAD total score (n=2925), National Health and Nutrition Examination Survey, 2011–2014.

| **Outcome** | OR (95%CI) P value | | |
| --- | --- | --- | --- |
|  | Model 1 | Model 2 | Model 3 |
| **Composite z-score** |  |  |  |
| GNRI (continuous) | 0.99 (0.98, 1.00) 0.003 | 0.99 (0.98, 1.00) 0.005 | 0.99 (0.99, 1.00) 0.007 |
| GNRI (quartile) |  |  |  |
| Q1 | Reference | Reference | Reference |
| Q2 | 0.73 (0.56, 0.97) 0.029 | 0.75 (0.56, 1.01) 0.059 | 0.76 (0.55, 1.05) 0.096 |
| Q3 | 0.58 (0.43, 0.79) < 0.001 | 0.59 (0.42, 0.81) 0.001 | 0.56 (0.38, 0.81) 0.002 |
| Q4 | 0.51 (0.36, 0.82) 0.004 | 0.51 (0.36, 0.86) 0.009 | 0.50 (0.33, 0.85) 0.008 |
| P for trend | < 0.001 | < 0.001 | < 0.001 |
| **DSST** |  |  |  |
| GNRI (continuous) | 0.99 (0.99, 1.00) 0.023 | 0.99 (0.99, 1.00) 0.051 | 0.99 (0.99, 1.00) 0.061 |
| GNRI (quartile) |  |  |  |
| Q1 | Reference | Reference | Reference |
| Q2 | 0.81 (0.61, 1.07) 0.139 | 0.85 (0.63, 1.15) 0.303 | 0.86 (0.63, 1.18) 0.344 |
| Q3 | 0.70 (0.51, 0.94) 0.019 | 0.71 (0.51, 0.98) 0.040 | 0.71 (0.51, 1.01) 0.055 |
| Q4 | 0.56 (0.37, 0.83) 0.005 | 0.55 (0.35, 0.85) 0.008 | 0.59 (0.37, 0.93) 0.023 |
| P for trend | < 0.001 | < 0.001 | < 0.001 |
| **AFT** |  |  |  |
| GNRI (continuous) | 0.99 (0.99, 1.00) 0.004 | 0.99 (0.99, 1.00) 0.006 | 0.99 (0.99, 1.00) 0.061 |
| GNRI (quartile) |  |  |  |
| Q1 | Reference | Reference | Reference |
| Q2 | 0.76 (0.60, 0.97) 0.027 | 0.72 (0.55, 0.93) 0.012 | 0.73 (0.56, 0.93) 0.012 |
| Q3 | 0.64 (0.49, 0.84) 0.001 | 0.62 (0.47, 0.83) 0.001 | 0.63 (0.48, 0.83) 0.001 |
| Q4 | 0.54 (0.38, 0.79) 0.001 | 0.53 (0.36, 0.78) 0.001 | 0.50 (0.34, 0.74) < 0.001 |
| P for trend | < 0.001 | < 0.001 | < 0.001 |
| **CERAD total score** |  |  |  |
| GNRI (continuous) | 0.99 (0.98, 0.99) < 0.001 | 0.99 (0.98, 0.99) < 0.001 | 0.99 (0.98, 1.00) < 0.001 |
| GNRI (quartile) |  |  |  |
| Q1 | Reference | Reference | Reference |
| Q2 | 0.76 (0.59, 0.98) 0.034 | 0.78 (0.59, 1.02) 0.065 | 0.81 (0.62, 1.07) 0.132 |
| Q3 | 0.64 (0.49, 0.85) 0.002 | 0.64 (0.48, 0.87) 0.004 | 0.66 (0.49, 0.90) 0.009 |
| Q4 | 0.53 (0.35, 0.78) 0.002 | 0.54 (0.36, 0.83) 0.005 | 0.58 (0.37, 0.90) 0.015 |
| P for trend | < 0.001 | < 0.001 | < 0.001 |

95%CI, 95% confidence intervals.

Crude Model was adjusted for none.

Model 1 was adjusted for age, sex, race, education, marital status, PIR.

Model 2 was adjusted for age, sex, race, education, marital status, PIR, smoking status, drinking, work activity, depression, history of diseases ((hypertension, stroke, diabetes, Parkinson, CVD).

Q1 (Quartile 1): ≤ 110; Q2 (Quartile 2): 111 - 119; Q3 (Quartile 3): 120 - 128; Q4 (Quartile 4): > 128. * P < 0.05 was considered statistically significant.
